# Evaluation of short- and mid-term benefits of re-operative surgery in iatrogenic spondylolisthesis cases

**DOI:** 10.25122/jml-2025-0048

**Published:** 2025-03

**Authors:** Dana-Georgiana Nedelea, Diana Elena Vulpe, Serban Dragosloveanu, Ioan Cristian Stoica

**Affiliations:** 1 Carol Davila University of Medicine and Pharmacy, Bucharest, Romania; 2 Department of Orthopedics, Foisor Clinical Hospital of Orthopedics, Traumatology and Osteoarticular Tuberculosis, Bucharest, Romania

**Keywords:** iatrogenic spondylolisthesis, laminectomy, paraspinal muscle degeneration, body mass index, spinal biomechanics, patient outcome, imaging, surgical treatment

## Abstract

Laminectomy is a widely used surgical approach in patients with spinal canal stenosis but can result in biomechanical changes leading to iatrogenic spondylolisthesis. While some factors, such as surgical technique and achievement of spinal stability, are key contributors, patient-specific factors remain underexplored. Our study included 64 patients with diagnosed iatrogenic spondylolisthesis following previous spinal surgery. They were stratified into male and female groups, and clinical parameters such as the body mass index (BMI), fatty infiltration of the paraspinal muscles (assessed via Goutallier classification), pain scores (Visual Analogue Scale - VAS), and functional outcomes (Oswestry Disability Index - ODI) were analyzed pre-and postoperatively. The cohort consisted of 19 men and 45 women, with a mean age of 63.7 ± 10.82 years. There was a statistically significant difference in BMI, with higher values in women than men (30.16 ± 2.73 vs. 28.11 ± 2.71, *P* = 0.0078). Fatty infiltration of the paraspinal muscles was also more pronounced in women, with significant differences observed in Goutallier grades 2 and 3 (*P* = 0.007). While no differences were noted in surgery duration or hospital stay, males experienced greater intraoperative blood loss (*P* = 0.0442). Both groups had similar short- and mid-term pain and functional improvement, with no statistically significant differences in the VAS or ODI scores. In conclusion, patients with iatrogenic spondylolisthesis showed sex-based differences in BMI and fatty infiltration of the paraspinal muscles in patients. These factors did not influence short- and mid-term functional recovery but may play a key role in disease progression and surgical outcomes.

## INTRODUCTION

Laminectomy represents one of the most frequently performed surgical interventions for symptomatic spinal canal stenosis, with over 490.000 procedures done annually in the United States [1]. With adequate patient selection, proper pre-operative spine assessment, and limited and correctly done decompression, laminectomy offers good long-term outcomes and excellent patient satisfaction. However, in some cases, a laminectomy can modify the normal spinal biomechanics, which leads to spinal segment instability and, thus, postoperative iatrogenic spondylolisthesis [2]. First described by Wiltse and White in 1976 [3], iatrogenic spondylolisthesis refers to the anterior or posterior slippage of a vertebral body as a complication of spinal surgery, most commonly after decompressive laminectomy. Iatrogenic spondylolisthesis has seen an increase in incidence over the years, with a frequency reported in the literature between 0 and 63% [4].

The current management strategies range from conservative treatment to re-operative stabilization, which typically involves spinal fusion with or without instrumentation and is aimed at restoring spinal alignment [5].

With growing socio-economical costs because of re-interventions and prolonged physical therapy and rehabilitation programs, iatrogenic spondylolisthesis remains controversial. Moreover, a difference based on sex and body mass index of patients, correlated with the fatty infiltration of the paravertebral muscles of the spine, is an understudied topic and may demonstrate relevance as these factors play a crucial role in the progression of spondylolisthesis.

## MATERIAL AND METHODS

### Study protocol

Our retrospective study included 64 patients admitted between November 2016 and December 2024 at Foisor Clinical Hospital of Orthopedics, Traumatology, and Osteoarticular TB, diagnosed with iatrogenic spondylolisthesis. All patients included in this study underwent surgery and had a history of previous spinal intervention and imagistic findings of spondylolisthesis. Patients reported to our ambulatory care unit for back pain or/and leg pain associated with imaging findings of spondylolisthesis. A thorough clinical examination was then performed by the same spine specialist to minimize bias.

If no neurologic impairment was noted, patients were included in a three-month protocol of conservative treatment, including lifestyle changes, 2 weeks of anti-inflammatory and pain medication every month, and inclusion in a physical therapy and rehabilitation program. Back pain and leg pain were assessed with the Visual Analogue Scale (VAS) [6], and the Oswestry Disability Index (ODI) [7] was also obtained at the beginning of conservative treatment and compared with scores obtained at the end of the three months. If a difference of less than 3 points on the VAS scale was observed, or if the patient reported unsatisfactory results following the 3-month rehabilitation protocol, they were referred for surgical treatment and included in this study. Conversely, if neurological impairment was detected during the initial clinical examination, patients were immediately referred for surgical treatment and included in this study.

### Initial evaluation

Upon admission to the hospital, all patients were assessed by the same team of spinal specialists. Demographic information gathered included gender, age, sex, body weight, height, and body mass index (BMI). Additional details regarding previous surgeries were also collected, including the location of the spondylolisthesis, the time since the initial spinal intervention, and the severity of the slippage as classified by Meyerding’s system [8], as well as an assessment of the fatty infiltration of paraspinal muscles according to the Goutallier classification [9]. The spinal specialist evaluated every patient's lower back and leg pain preoperatively using the Visual Analogue Scale (VAS), and the Frankel classification was used to assess neurological impairment [10]. The Oswestry Disability Index (ODI) was used to determine how much pain impacts the patient’s daily activities and social life. The low-back outcome scale formulated by Greenough and Fraser is more extensive than the Oswestry Disability Index, including 13 items such as pain, employment, sport, active social activities, overall analgesics consumption, and activities of daily living were also assessed in this study [11].

### Outcome assessment

All patients underwent surgery, and the following data were recorded: total surgical time (from anesthesia induction to the placement of the last thread) in minutes, intraoperative blood loss in milliliters, length of hospital stay in days, number of levels fused, reduction of spondylolisthesis in millimeters, grade of reduction according to Meyerding’s classification, and postoperative low back pain and leg pain as assessed by the VAS. Postoperative neurological function was evaluated using the Frankel classification. Follow-up included clinical and radiological examinations six months after surgery, with a reassessment of low back and leg pain using the VAS, the Oswestry Disability Index, and the low-back outcome scale by Greenough and Fraser. Implant failure, whether involving the rods or screws, was also evaluated.

### Imaging diagnosis and classification

At admission, blood samples were drawn from all patients to prepare them for surgery. Plain radiographs of the spine and MRI were performed, centered on the affected levels. All patients routinely underwent anteroposterior (AP) and lateral (LL) spine radiographs, along with specialized projections that assess spinal instability functionally, conducted in our Department of Radiology and Medical Imaging. These radiographs were performed using a DigitalDiagnost R3.1 machine (Philips Medical Systems Nederland B.V., Amsterdam, The Netherlands). The examinations were done in a standing position to highlight segmental spinal instability. The specialized functional tests for lumbar spine instability included lumbar spine flexion and extension lateral views, which are performed after the standard lateral view of the lumbar spine. In these tests, the patient is instructed to 'bend forward' as much as possible for the flexion lateral view or 'lean back' for the extension lateral view. Post-surgery, AP, and LL spine radiographs were repeated. The digital images were reviewed for quality and then archived. All images were stored and accessed via the hospital's picture archiving system and viewed using a dedicated radiology monitor and software.

All patients in the study received an MRI of the lumbar spine before surgery. All MRI examinations included sagittal T1- and T2-weighted sequences and an axial T2-weighted sequence on which the qualitative measurements of paraspinal muscles were performed. The MRI images were provided by external suppliers and were performed before admission to the hospital.

Measurements were conducted by the same spinal specialist using the same imaging software to minimize software bias. The radiographic images stored in the hospital’s archiving and communication system (PACS) were analyzed using MediCAD Hectec GmbH, Altdorf, Germany.

**Figure 1:**
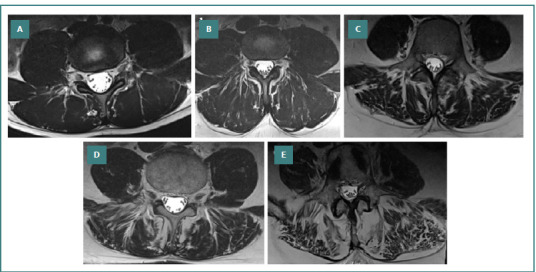
The Goutallier classification of overall qualitative fatty infiltration of the paraspinal muscles was exemplified in five patients from our cohort on axial T2-weighted sequences. A, grade 0, no visible fatty infiltration of the paraspinal muscles; B, grade 1, a few fatty streaks are visible within the paraspinal muscles; C, grade 2, less than 50% of paraspinal muscle atrophied; D, grade 3, an equal amount of muscle and fat is present within the paraspinal muscles; E, grade 4, more than 50% of the paraspinal muscle is fatty infiltration.

The qualitative measurements of the fatty infiltration found in the paraspinal muscles were performed according to the Goutallier classification system on axial T2-weighted sequence MRI. These were performed at the level of the spondylolisthesis, one level above and one level below. Overall, qualitative fatty infiltration was used as an average Goutallier for all three segments mentioned above. The grading comprises grade 0 – no visible fatty infiltration, grade 1 – a few fatty streaks within the paraspinal muscles, grade 2 – less than 50% muscle atrophy, grade 3 – the same amount of fat and muscle within the paraspinal muscles, and grade 4 – greater than 50% fatty muscle atrophy (Figure 1A-E) [9].

### Surgical approach

All patients in this study were submitted for surgical treatment consisting of Posterior Lumbar Interbody Fusion (PLIF) and were operated on by the same spinal surgical team within the time interval 2016–2024. Patients were positioned in a prone position on the operating table. A midline approach centered on the affected spine region was performed, with bilateral muscle strip dissection and fibrous tissue release from the previous spine surgery. Pedicle screw fixation was performed according to the preoperative planning. A laminectomy at the appropriate level was then performed, with care to the release of fibrotic tissue from previous surgery, and access to the intervertebral disc space was acquired. An interbody cage insertion was performed under fluoroscopic control. Then, rods were implanted, and a final verification of the instrumentation was performed.

### Statistics and analytics

The statistical analysis was performed using Stata/IC (version 16, StataCorp, College Station, TX, USA). Data were presented as mean ± standard deviation (SD) for continuous variables and frequencies with percentages for categorical variables. Data distribution was tested for normality, and the *t*-test was used to compare continuous variables between male and female patients, while Fisher’s exact test was used for categorical variables. Statistical significance was set at *P* < 0.05.

## RESULTS

The analyses comprised 64 patients, 45 women and 19 men, aged between 36 and 80, with a mean value of 63.7 ± 10.82 years old. In most cases, the spondylolisthesis was located at L4-L5 and L5-S1. Table 1 presents an overview of the demographic characteristics of the studied population and the anatomical variations of the affected levels of spondylolisthesis.

**Table 1:** Demographic characteristics of the studied population and the anatomical variation and distribution of the affected levels of spondylolisthesis

Parameter	Men	Women
Cohort	19	45
Mean age (years)	64.32 ± 11.18	63.44 ± 10.79
Body Mass Index (BMI)	28.11 ± 2.71	30.16 ± 2.73
**Location of spondylolisthesis**		
L2-L3	0	1
L3-L4	0	2
L4-L5	10	22
L5-S1	9	20

* data presented as mean value ± standard deviation

We paired the cohort of patients based on sex—men and women. At the time of surgery, the average age was 64.32 ± 11.18 years for men and 63.44 ± 10.79 years for women. A statistically significant difference (*P* = 0.0078) was observed between the two groups, as men had lower BMI (28.11 ± 2.71) than women (30.16 ± 2.73). No correlation was found between the location of the spondylolisthesis and BMI.

Regarding the fatty infiltration of paraspinal muscles as classified by Goutallier, women had higher fatty degeneration than men, with a statistically significant difference (*P* = 0.007) for Goutallier grades 2 and 3, as presented in Table 2.

**Table 2:** Number of cases classified according to the degree of fatty infiltration in the paraspinal muscles in each group

Goutallier classification	Men	Women	*P* value
Grade 1	17	17	0.0007
Grade 2	2	20	
Grade 3	0	8	

In terms of surgery and hospital stay, no difference was noted in the duration of surgery and hospital length of stay. A slight significant difference was distinguished in the blood loss measured during the surgery, as men had greater intra-operative blood loss than women (*P* = 0.0442). These data are presented in Table 3.

**Table 3:** Intra-operative data and the length of stay in the hospital

Parameter	Men	Women	*P* value
Duration of surgery (mins)	186.32 ± 27.53	170.89 ± 33.83	0.0842
Intraoperative blood loss (mL)	735.26 ± 141.87	647.78 ± 160.95	0.0442
Hospitalization (days)	6.32 ± 0.82	6.87 ± 3.15	0.2802

* mins.= minutes, mL.= mililiters, data presented as mean value ± standard deviation.

Regarding the short-and mid-term improvement of pain and function, no statistically significant differences were noted between the two groups (Table 4).

**Table 4:** Outcome scores obtained by the patients at the 6-month follow-up

Parameter	Men	Women	*P* value
Low back pain reduction	-0.15 ± 1.74	0.53 ± 1.61	0.1318
Leg pain reduction	-0.47 ± 2.29	0.07 ± 1.78	0.3128
ODI reduction	15.82 ± 9.47	13.09 ± 8.90	0.2715
GF scale improvement	19.05 ± 2.93	19.53 ± 3.18	0.5743

* low back pain and leg pain reduction were calculated as a difference in score on the VAS at admission and six months follow-up for back and lumbar pain, respectively. ODI, Oswestry Disability Index, GF scale, Greenough, and Fraser scale were measured at admission. Data presented as mean ± standard deviation.

## Discussion

The incidence of iatrogenic spondylolisthesis following decompression in the current literature is extremely variable, cited between 0 and 63% [4,12]. This variability may come from the fact that no difference has been made between individuals with prior spondylolisthesis before decompression versus individuals with no prior biomechanical impairment. Newer studies try to define the distinction between decompression alone and decompression and fusion in patients with spondylolisthesis and the need to proceed with one or another. In a randomized controlled trial meant to compare the effectiveness of adding a lumbar fusion for patients undergoing decompressive laminectomy for spinal stenosis associated with symptomatic grade I lumbar degenerative spondylolisthesis, results showed that the addition of fusion was correlated with clinical improvement in overall quality of life [13]. Many spine surgeons from all over the world consider that fusion associated with decompression is a more effective treatment for slippage and dynamic instability in cases associated with spondylolisthesis [14]. However, in our country, we still submit for surgery numerous patients who had undergone laminectomy or other spine interventions in different facilities and present with symptomatic spondylolisthesis at various time intervals between the first spinal surgery.

The role of posterior osseoligamentous structures of the spine is extremely important, and biomechanical studies have shown that segmental mobility increases after the disruption of the posterior osseoligamentous structures after bilateral laminectomy [15]. Paraspinal muscle integrity is essential for maintaining overall spinal alignment, and fatty infiltration and atrophy of these muscles can contribute to changes in normal lumbar lordosis and sacral-vertebral angle [16].

Medical imaging plays an essential role in the diagnosis and management of patients with iatrogenic spondylolisthesis. Dynamic radiographs are valuable in detecting segmental instability, while MRI provides detailed information on the neural structures and paravertebral soft tissues [17]. Imaging supports decision-making, preoperative planning, and follow-up, and it plays a critical role in assessing vertebral alignment and arthrodesis [18].

Worldwide, the prevalence of sarcopenia can vary from 9.9% to 40.4% [19]. Moreover, the prevalence of sarcopenia increases with age; from age 30, the muscle mass decreases by around 3% to 8% per decade, with an accelerated decrease over 60 years old [20,21]. Furthermore, 69% of our patients are women with an average age of 63.44 ± 10.79 years old, at menopause or within the menopausal transition, affected by the hormonal changes that may occur at this specific age, especially related to the decrease in estrogen levels. We found a statistically significant difference between men and women in our study regarding the amount of fatty infiltration and degeneration of paraspinal muscles, with higher grades on the Goutallier classification achieved by women. This may be influenced by the higher BMI in the women group, but also, as studied recently, an effect of the musculoskeletal syndrome of menopause, which includes sarcopenia or the loss of lean muscle mass related to estrogen decrease and other menopause-induced modifications [22].

According to our study, women had a significantly higher BMI than men despite being younger on average. BMI, an anthropometric index developed in 1832, has been shown to inadequately assess body fat and muscle mass. Alternative measures, such as waist circumference, waist-to-hip ratio, and body shape index, may offer a more accurate evaluation [23]. Unfortunately, our retrospective study would not permit us to make any changes to the use of other anthropometric indexes to define a patient’s body fat and/or muscle mass percentage. These findings would be beneficial for further assessment in future prospective studies.

In our study, no correlation was found between BMI and the location of spondylolisthesis. However, several studies emphasize the role of weight and BMI in spondylolisthesis. For instance, Schuller *et al*. reported a mean BMI of 28.2 in their spondylolisthesis population, noting that 71.4% of affected individuals were overweight or obese, and identified a correlation between BMI and L4-L5 slippage associated with sagittally oriented facet joints [24]. Moreover, Ebstein *et al*. found that higher BMI is correlated with surgery failure in patients with degenerative spondylolisthesis [25]. Additionally, other research suggests that patients with an overweight BMI have a 6.089-fold increased risk of developing lumbosacral spondylolisthesis compared to those with a normal BMI [26].

Regarding follow-up and short- and mid-term results, there were no statistically significant differences in scores at six months of follow-up between the two groups. The Oswestry Disability Index showed no difference in patient satisfaction between groups at the six-month follow-up. Similar results were also noted in the literature for long-term follow-up, although no study has focused on iatrogenic-only spondylolisthesis [27,28].

From an economic point of view, iatrogenic spondylolisthesis implies higher costs related to more complex surgeries, longer rehabilitation, extended hospital stays, and possibly loss of productivity, therefore impacting society and contributing to higher healthcare costs [29].

With a continuous increase in the prevalence of spinal surgery, it is expected that iatrogenic spondylolisthesis will also be more frequent [30]. Future studies should focus on improving diagnostic accuracy using advanced imaging methods and predictive models that analyze various patient-specific factors; this would allow for creating clinical algorithms for the selection of candidates that most benefit from re-operation, therefore reducing the rate of revision surgery and improving the patient outcomes on the long term [31]. Moreover, the use of artificial intelligence, intraoperative navigation platforms, or 3D imaging can improve surgical precision and limit the risk of postoperative instability [32].

This study has some limitations. It is a single-center retrospective study, with collected data from the hospital’s archives limited to a few demographic data collected. Secondly, the limited sample size is an important factor, although this study focuses on iatrogenic-only spondylolisthesis that requires surgery.

## CONCLUSION

All patients are influenced by the ensuing age-related sarcopenia, but the results of our study suggest that women are strongly affected by the menopausal-hormonal alterations and become more affected by this change in lean muscle homeostasis. This parameter would have a major impact on all musculoskeletal disorders, but it plays a critical part in iatrogenic spondylolisthesis, where women are more likely to have a higher BMI and higher grade of fatty infiltration of the paraspinal muscles, albeit being younger at the time of surgery than men affected by this disease.

## Data Availability

Further data is available from the corresponding author upon reasonable request.
